# 肺癌合并间质性肺疾病49例临床特征分析

**DOI:** 10.3779/j.issn.1009-3419.2026.106.25

**Published:** 2026-08-20

**Authors:** Jing ZHAO, Lihua ZHAO, Xiaoxing GAO, Xiaoyan LIU, Yan XU, Minjiang CHEN, Wei ZHONG, Mengzhao WANG

**Affiliations:** 1 100730 北京，中国医学科学院北京协和医学院，北京协和医院呼吸与危重症医学科; 1 Department of Pulmonary and Critical Care Medicine, Peking Union Medical College Hospital, Peking Union Medical College,Chinese Academy of Medical Sciences, Beijing 100730, China; 2 100038 北京，首都医科大学附属复兴医院呼吸与危重症医学科; 2 Department of Pulmonary and Critical Care Medicine,Fuxing Hospital, Capital Medical University, Beijing 100038, China

**Keywords:** 肺肿瘤, 间质性肺疾病, 普通型间质性肺炎, 临床特征, Lung neoplasms, Interstitial lung disease, Usual interstitial pneumonia, Clinical characteristics

## Abstract

**背景与目的:**

肺癌合并间质性肺疾病（lung cancer combined with interstitial lung disease, LC-ILD）的共病患者比例高，预后不佳，且没有标准治疗方案。本研究拟总结LC-ILD患者临床特征及治疗效果，并探讨不同的肿瘤治疗方式下出现ILD明显进展的相关因素。

**方法:**

收集2018年1月至2024年12月于北京协和医院呼吸与危重症医学科住院诊治的49例LC-ILD患者的临床特征、肿瘤治疗方式及生存随访情况；观察不同治疗方式的疗效及不良反应，同时进行生存分析，以及分析导致ILD明显进展的可能因素。

**结果:**

LC-ILD以普通型间质性肺炎（usual interstitial pneumonia, UIP）型多见，肿瘤好发于纤维化区域，中位无进展生存期（progression-free survival, PFS）在晚期非小细胞肺癌（non-small cell lung cancer, NSCLC）为7.0个月，在小细胞肺癌（small cell lung cancer, SCLC）为4.0个月。36例完成治疗和随访者中有10例出现影像学间质病变进展，其中4例死亡，*Logistic*回归分析显示放疗与影像学间质病变进展有明显关联（*P*<0.05）。

**结论:**

对于LC-ILD患者，治疗前应进行包括胸部高分辨率计算机断层扫描、肺功能（弥散功能）在内的个体化评估，治疗策略需权衡疗效与安全性，尤其警惕放疗导致ILD进展的风险。

## Abstract (en)

**Background and objective:**

The patients of lung cancer combined with interstitial lung disease (LC-ILD) account for a high proportion, and their prognosis is poor. Currently, no standard treatment regimen had been established for this population. This study aimed to summarize the clinical characteristics and treatment outcomes of patients with LC-ILD, and to explore factors associated with marked ILD progression under different tumor treatment strategies.

**Methods:**

The clinical characteristics, tumor treatment, and follow-up data of 49 LC-ILD patients hospitalized in the Department of Pulmonary and Critical Care Medicine at Peking Union Medical College Hospital from January 2018 to December 2024 were collected. The efficacy and follow-up outcomes of different treatment modalities were observed; the survival analysis was performed, and the potential influencing factors for the significant progression of ILD were analyzed.

**Results:**

LC-ILD is most commonly of the usual interstitial pneumonia (UIP) pattern, with tumors frequently located in fibrotic regions. The median progression-free survival (PFS) was 7.0 months for advanced non-small cell lung cancer (NSCLC) and 4.0 months for small cell lung cancer (SCLC). Of the 36 patients with completed follow-up and treatment, 10 showed radiographic progression of interstitial lesions, with 4 deaths occurring in this subgroup. *Logistic *regression analysis indicated that radiotherapy was significantly correlated radiological progression of ILD (*P*<0.05).

**Conclusion:**

For patients with LC-ILD, individualized evaluation including chest high resolution computed tomography and pulmonary function test (particularly diffusing capacity) should be performed before initiating therapy. The therapeutic approach should balance efficacy and safety, and special attention should be given to the risk of radiological progression of ILD associated with radiotherapy.

近年来肺癌合并间质性肺疾病（lung cancer combined with interstitial lung disease, LC-ILD）的情况越来越受到关注。肺癌可继发于ILD或者共同危险因素导致两者同时发生，肺癌患者中合并ILD比例高达5.8%-15.2%^[1]^。针对晚期肺癌的治疗除了化疗、放疗外，近年来有了突破性进展，分子靶向药物、免疫检查点抑制剂显著延长患者生存期，但这些治疗手段对于ILD患者属相对禁忌，因为这些药物本身会引起间质性肺炎，合并ILD患者风险更高^[2,3]^。肺癌与ILD这两种预后不佳疾病共存的患者，目前没有标准治疗方案，而且治疗的获益和风险尚不明确。因此，这类患者的临床特征、治疗效果和预后情况，乃至个体化治疗策略，仍亟待解决。本研究旨在总结LC-ILD患者的临床特征、治疗效果以及有无出现ILD进展，并探讨导致ILD进展的相关因素，为今后对这类患者制定个体化治疗策略提供参考。

## 1 资料与方法

### 1.1 研究对象

利用电子病历系统收集2018年1月至2024年12月于北京协和医院呼吸与危重症医学科住院诊治的肺部恶性肿瘤同时合并胸部高分辨率计算机断层扫描（high resolution computed tomography, HRCT）显示存在双肺间质性改变的患者，共128例。本回顾性研究获得北京协和医院伦理委员会批准（No.K4607）。因回顾性研究属性，患者知情同意书豁免。

研究对象纳入标准：肺癌经组织病理学确诊；ILD的诊断分类参照美国胸科协会和欧洲呼吸学会2025年颁布的ILD分类共识^[4]^中胸部HRCT特征为间质模式，同时依据2018年颁布的特发性肺纤维化（idiopathic pulmonary fibrosis, IPF）诊断指南^[5]^，结合病史、血清学自身抗体检测，分为：普通型间质性肺炎（usual interstitial pneumonia, UIP）、可能UIP型、非UIP型。排除标准：（1）原发肿瘤非肺癌者；（2）感染、药物导致的ILD，包括免疫检查点抑制剂相关肺炎（checkpoint inhibitors pneumonia, CIP）；（3）放疗引起的放射性肺炎（radioactive pneumonia, RP）；（4）慢性阻塞性肺病（chronic obstructive pulmonary disease, COPD）合并间质性肺异常（interstitial lung abnormalities, ILA）；（5）肺纤维化合并肺气肿（combined pulmonary fibrosis and emphysema, CPFE）；（6）HRCT特征为肺泡填充模式ILD。收集的128例患者中，经过严格筛选后排除资料不全16例，原发肿瘤非肺癌2例，CIP 23例，RP 3例，CIP合并RP 2例，小分子靶向药物所致的间质性肺炎9例，感染导致ILD 8例，COPD合并ILA 14例，CPFE 2例，最终入组49例。

### 1.2 研究方法

回顾性收集病例资料，包括：人口学资料、ILD类型、ILD之于肿瘤发现的时序、肺癌的病理类型、分期、影像学特征、肺功能、治疗方式等。ILD分类是依据胸部HRCT主要特征，由2名呼吸与危重症医学科高年资医师来判定，对于不一致的结果，由第3名呼吸与危重症医学科高年资医师最终判断。如生存情况不详需对患者及家属进行电话随访，获取资料包括：无事件生存期（event free survival, EFS）、无进展生存期（progression-free survival, PFS）以及总生存期（overall survival, OS）。起点为首次治疗日，终点为随访期间复查影像学临床判定为疾病复发、进展或死亡的时间。本研究截止时间为2025年2月，数据截止日仍未发生终点事件的患者，以末次随访日作为删失时间。

研究筛选出在治疗过程中影像学显示间质病变明显进展的LC-ILD患者，并进一步分析与其相关的因素。影像学进展，是指在系列HRCT随访过程中，ILD受累范围增加超过10%，按照进展性肺纤维化的评判标准“HRCT中纤维化范围增加”^[6]^，并结合本研究实际情况确定的操作性定义。由2名呼吸与危重症医学科高年资医师通过视觉半定量独立做出判断，采用Kappa一致性检验来评估该2名医师判断一致性，两者Kappa值为0.78（Kappa值>0.75为一致性良好）。对于判断不一致的病例，由第3名高年资医师进行判断，最终确定是否进展。

### 1.3 统计方法

数据分析采用SPSS 24.0软件，计数资料用例数和百分比表示，两组比较应用卡方检验；计量资料如呈现正态分布用均数±标准差表示，两组比较采用t检验。生存分析采用*Kaplan-Meier*法，对是否出现间质病变进展的两组先进行单因素*Logistic*回归分析，然后将*P*<0.1的变量纳入多因素*Logistic*回归分析，以进一步校正混杂因素，并计算优势比（odds ratio, OR）及其95%CI。*P*<0.05为差异具有统计学意义。

## 2 结果

### 2.1 临床基线资料

49例患者中男性43例，女性6例，平均年龄为（67.4±5.7）岁。吸烟者包括正在吸烟及既往吸烟者共41例，其中吸烟指数>400者占92.7%。合并ILD种类，UIP型为32例[29例特发，3例结缔组织病（connective tissue disease, CTD）相关的UIP：2例类风湿关节炎，1例抗中性粒细胞胞浆抗体（anti-neutrophil cytoplasmic antibody, ANCA）相关血管炎]，可能UIP型为3例，非UIP型为14例（7例特发，7例CTD有关：皮肌炎、ANCA相关血管炎各1例、未分类CTD 5例）。两种疾病有26例为同时诊断，23例在肺癌诊断前已诊断为ILD。肺癌病理类型包括腺癌、鳞癌、腺鳞癌、小细胞肺癌（small cell lung cancer, SCLC）及混合性小细胞癌。肿瘤原发灶-淋巴结-转移（tumor-node-metastasis, TNM）分期中以III和IV期为主（81.7%）；原发病灶影像学多表现为孤立肿块或结节影（周围型），位于下叶最多（63.3%）；多见于网格影或蜂窝影区域（83.7%）（表1）。

**表1:** LC-ILD患者的基线资料（*n*=49）

Index	*n* (%)
Age (yr)	
<65	15 (30.6)
65-74	30 (61.2)
≥75	4 (8.2)
Gender	
Male	43 (87.8)
Female	6 (12.2)
Smoking status	
Never smoking	8 (16.3)
Former smoking	20 (40.8)
Current smoking	21 (42.9)
ILD subtype	
UIP^#^	32 (65.3）
Probable UIP	3 (6.1)
Non-UIP^&^	14 (28.6)
Timing of ILD relative to lung cancer	
ILD precedes lung cancer	23 (46.9)
ILD and lung cancer concurrently 26 (53.1)	
Histology	
Adenocarcinoma	20 (40.8)
Squamous cell carcinoma	17 (34.7)
Adenosquamous carcinoma	1 (2.0)
SCLC	9 (18.4)
Mixed small cell carcinoma	2 (4.1)
TNM stage	
I	6 (12.2)
II	2 (4.1)
III	19 (38.8)
IV	21 (42.9)
Unknown	1 (2.0)
Primary tumor location	
Upper lobe	12 (24.5)
Lower lobe	31 (63.3)
Hilar	3 (6.1)
Not evaluable	3 (6.1)
Tumor in fibrotic area	
Yes	41 (83.7)
No	8 (16.3)

^#^including 29 cases of idiopathic UIP and 3 cases of CTD-UIP; ^&^including 7 cases of idiopathic non-UIP and 7 cases of CTD-ILD. ILD: interstitial lung disease; UIP: usual interstitial pneumonia; SCLC: small cell lung cancer; TNM: tumor-node-metastasis; CTD: connective tissue disease.

22例完成了包含弥散功能的肺功能检查，均存在中重度弥散功能障碍，同时合并限制性通气功能障碍者14例；主要指标结果包括：第1秒用力呼气容积占预计值百分比（forced expiratory volume in the first second, percentage of predicted value, FEV_1_%pred）为（83.13±20.47）%，肺总量占预计值百分比（total lung capacity, percentage of predicted value, TLC%pred）为（74.37±14.22）%，一氧化碳弥散量占预计值百分比（diffusing capacity of the lungs for carbon monoxide, percentage of predicted value, DLCO%pred）为（49.17±13.60）%。

### 2.2 治疗及随访

#### 2.2.1 治疗方法

非小细胞肺癌（non-small cell lung cancer, NSCLC）共38例。1例未分期者接受局部治疗。14例I-III期可手术或潜在可切除者：2例肺部间质病变严重仅行局部治疗；其余12例均接受了根治性手术治疗，其中2例单纯手术，1例新辅助化疗联合免疫，1例辅助靶向，8例辅助化疗（其中2例序贯辅助放疗）。23例III期不可切除和IV期患者：2例因肺部间质病变严重行局部治疗联合安罗替尼或奥希替尼[存在表皮生长因子受体（epidermal growth factor receptor, EGFR）21 L858R突变]；其余21例接受了全身化疗，其中6例联合免疫治疗。

SCLC共11例，局限期5例中1例因TNM分期为IB期行手术及辅助化疗；其余4例给予化疗，其中2例联合序贯放疗；广泛期6例均接受化疗，其中2例给予序贯肺部病变姑息放疗。

#### 2.2.2 治疗结局

13例治疗未满4个周期且未再就诊，无法进行疗效评估；其余36例完成后续治疗或门诊随访，中位随访时间为13.0个月（范围：3.0-49.0个月）。

手术治疗13例（NSCLC为12例，SCLC为1例），12例NSCLC的中位随访时间为18.5个月（范围：5.0-46.0个月），*Kaplan-Meier*分析（图1A）显示6和12个月的EFS率均为 66.7%，24个月为55.6%，中位EFS未达到。1例SCLC术后行辅助化疗3个周期，因不良反应未再就诊治疗。不可切除的局部晚期或IV期NSCLC为14例，不可手术SCLC为9例。治疗4个周期评估达到确认部分缓解（partial response, PR）或完全缓解（complete response, CR）者共9例，NSCLC的客观缓解率（objective response rate, ORR）为35.7%（5/14），SCLC为44.4%（4/9）。*Kaplan-Meier*分析（图1B）显示，NSCLC的中位PFS为7.0个月（范围：2.0-46.0个月），SCLC为4.0个月（范围：2.0-9.0个月）；NSCLC的6个月PFS率仅为51.4%，SCLC仅为27.8%；NSCLC的12个月PFS率为17.1%。

**图1:**
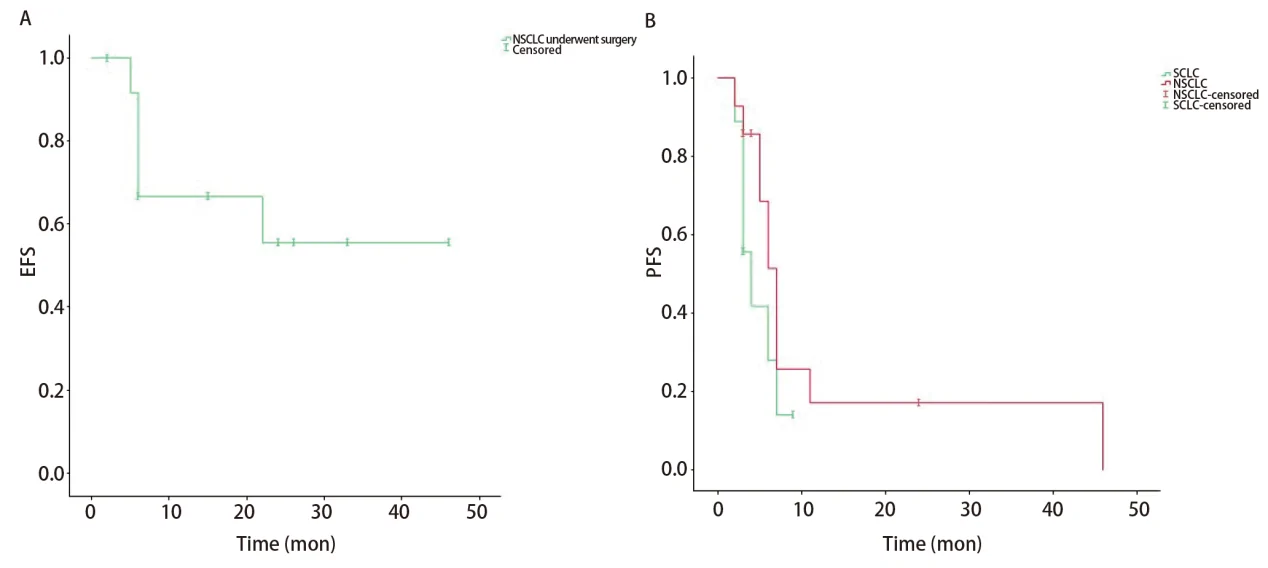
Kaplan-Meier分析。A：手术治疗的NSCLC患者（n=12）EFS的Kaplan-Meier曲线；B：不可切除的局部晚期或IV期的NSCLC（n=14）和SCLC（n=9）患者PFS的Kaplan-Meier曲线。

接受免疫治疗为7例，1例为术前新辅助化疗联合免疫，出现免疫性肠炎（2级），停药后好转，之后再次免疫治疗，出现CIP（4级），激素治疗好转后手术治疗，术后随访33个月未复发。6例为IV期NSCLC接受一线化疗联合免疫治疗。1例治疗1个周期后未再就诊及随访；其余5例的ORR为40.0%（2/5），中位PFS为7个月；其中3例均出现CIP（2例3级，停药并予激素治疗；1例2级，停药处理），均经治疗后好转。

#### 2.2.3 影像显示间质病变进展的发生情况

36例完成后续治疗或随访者中，10例出现影像学提示间质病变不同程度的进展，其中4例死亡（3例为治疗6个月内，1例为治疗7个月左右），其余6例间质病变进展，多数发生在治疗的6-12个月，未治疗，患者症状持续，肺功能较前减退。对导致间质病变进展的因素先后进行单因素和多因素*Logistic*回归分析（表2），结果显示放疗为影响因素（*P*=0.010）。针对不同化疗方案，结果显示使用紫杉类化疗方案导致间质病变进展的占比为14.3%（1/7），而使用非紫杉类药物导致的占36.0%（9/25），后者有增高趋势，但未达统计学差异（*P*=0.293）（表2）。

**表2:** 肺部间质病变进展与否的两组患者临床特征比较

Item	No progression (*n*=26)	Progression (*n* =10)	Univariate analysis			Multivariate analysis	
	[*n* (%)]	[*n* (%)]	OR (95%Cl)	*P*		OR (95%Cl)	*P*
Age (yr)	68.8±5.9	64.4±6.0	0.88 (0.76-1.01)	0.065		0.86 (0.73-1.02)	0.090
Sex			0.52 (0.07-3.71)	0.516			
Male	23 (88.5)	8 (80.0)					
Female	3 (11.5)	2 (20.0)					
Histology			0.36 (0.07-1.76)	0.206			
NSCLC	21 (80.8)	6 (60.0)					
SCLC and mixed	5 (19.2)	4 (40.0)					
TNM stage			1.46 (0.31-6.98)	0.637			
Non IV stage	16 (61.5)	7 (70.0)					
IV stage	10 (38.5)	3 (30.0)					
ILD subtype			1.78 (0.31-10.32)	0.521			
UIP*	18 (69.2)	8 (80.0)					
Others	8 (30.8)	2 (20.0)					
Tumor in fibrotic area			0.30 (0.05-1.86)	0.198			
Yes	23 (88.5)	7 (70.0)					
No	3 (11.5)	3 (30.0)					
Corticosteroid therapy for ILD			1.20 (0.20-7.25)	0.842			
Yes	6 (23.1)	2 (20.0)					
No	20 (76.9)	8 (80.0)					
Antifibrotic therapy for ILD			0.86 (0.17-4.29)	0.854			
Yes	7 (26.9)	3 (30.0)					
No	19 (73.1)	7 (70.0)					
Surgery			1.24 (0.26-5.97)	0.793			
Yes	9 (34.6)	3 (30.0)					
No	17 (65.4)	7 (70.0)					
Radiotherapy			0.04 (0.00-0.42)	0.007		0.03 (0.00-0.45)	0.010
Yes	1 (3.8)	5 (50.0)					
No	25 (96.2)	5 (50.0)					
Chemotherapy^#^			0.30 (0.03-2.87)	0.293			
Taxanes	6 (27.3)	1 (10.0)					
Non-taxanes	16 (72.7)	9 (90.0)					

*including 23 cases of idiopathic UIP and 3 cases of CTD-UIP. ^#^Among the 36 patients with complete follow-up data, 4 did not receive chemtherapy while the remaining 32 received chemtherapy. OR: odds ratio; CI: confidence interval.

## 3 讨论

本回顾性研究显示LC-ILD以老年男性、吸烟者为主，且合并ILD以UIP型多见，这与既往研究^[7]^结果一致。另外，本研究也显示肺癌好发于纤维化区域，且下叶更常见，而纤维化区域本身也是下叶、胸膜下显著，提示LC和ILD有相似的发生机制，包括上皮细胞反复损伤修复异常、免疫细胞失调以及基因突变（如*TP53*）等。LC-ILD病理类型以腺癌居多，其次为鳞癌。LC-ILD患者发现或确诊肿瘤时大多为中晚期，可能与合并ILD时症状易被忽视或影像学上难以甄别有关。本研究显示晚期NSCLC-ILD的ORR和中位PFS分别为35.7%和7.0个月，与Asahina等^[8]^的研究结果类似（分别为55.6%和5.3个月）。SCLC-ILD的ORR和中位PFS分别为44.4%和4.0个月，与Shirasawa等^[9]^的研究结果中ORR为64.5%和中位PFS为5.1个月类似。2025年LC-ILD国内专家共识^[10]^指出，早期肺癌应充分进行围术期风险评估，权衡获益与风险，通过多学科协作诊疗（multi-disciplinary treatment, MDT）讨论决定手术方式及切除范围。本研究中12例NSCLC接受了根治性手术，预后良好，24个月的EFS率为55.6%，中位EFS未达到，提示早期LC-ILD充分评估风险后，手术治疗效果最佳。

本研究多因素Logistic回归分析提示仅放疗与ILD进展有关，但因总样本量小，阳性事件仅10例，故该模型有一定局限性。近年来国外的一些研究^[11]^结果也显示放疗是导致间质病变进展的不利因素。10例发生间质病变进展的患者，其中占比最高的ILD为UIP型（8/10），另外2例中1例为可能UIP型，这在一定程度上反映了UIP型ILD发生病变进展的风险高。Kim等^[12]^研究证实肺癌合并IPF（均是UIP型）者进行放疗，造成病死率增加，故欧洲癌症研究与治疗组织建议该类患者避免应用放疗^[13]^。

合并ILD的NSCLC患者较少存在驱动基因。因此，化疗是此类患者主要治疗手段。Chen等^[14]^一项荟萃分析纳入7项研究共251例NSCLC-ILD患者，结果显示化疗造成ILD进展的发生率为8.47%。Asahina等^[8]^研究显示，白蛋白紫杉醇联合卡铂对于晚期NSCLC合并ILD患者有良好的安全性及有效率，ILD进展的发生率仅5.6%。Fujita等^[15]^的研究显示接受培美曲塞联合铂类治疗的患者中有12.5%出现ILD进展。本研究也显示，与使用紫杉醇化疗方案相比，使用非紫杉类化疗方案导致间质病变进展的发生率有增高趋势，但可能因例数少而未达统计学差异（*P*=0.293）。

免疫治疗已成为驱动基因阴性晚期NSCLC的标准疗法，因可导致CIP，故临床上一般避免在ILD患者中使用。研究^[16]^显示LC-ILD共病者发生CIP风险显著增加，尤其3级以上CIP发生。一项荟萃分析^[17]^发现合并ILD的NSCLC患者3级以上CIP发生率为12%。国内至今鲜有相关研究。本研究中7例患者使用免疫治疗，4例出现了CIP，发生率高达57%（4/7），其中1例2级，2例3级，1例4级，给予激素治疗均好转。

无论何种治疗手段均有不同程度发生ILD进展的风险，早期识别或治疗前评估至关重要。多项研究^[18,19]^总结出这类患者的预测因素，如肺功能（主要为DLCO）严重受损、生物标志物水平显著升高（如血清涎液化糖链抗原-6）、HRCT上纤维化程度、体能状况评分。本研究中10例发生影像不同程度进展，仅有2例完善了包括弥散功能在内的肺功能检查，一方面说明这些患者的肺功能很差无法耐受检查，另一方面可能是在治疗前对评估肺功能重视度不足。所以，治疗前全面个体化的评估尤为重要。

本研究中有10例患者在肿瘤治疗前或治疗中使用抗纤维化药物，对于是否引起影像进展方面未显示出关联。目前一些关于围术期应用吡非尼酮治疗LC-IPF的小样本研究^[20]^得出其可降低患者术后发生急性加重的风险，因此，在肿瘤治疗时尝试联合抗纤维化药物尼达尼布或吡非尼酮控制纤维化，还需更大样本及前瞻性研究进一步验证。

本研究有很多不足：（1）此项研究为单中心回顾性研究、样本量少、异质性较高，而且同一病理类型或分期的不同患者治疗方案也不尽相同，会存在选择偏倚；（2）研究结果可能存在混杂因素干扰，如难以充分排除合并病毒感染等因素，入选的ILD患者影像学以间质模式为主，排除肺泡填充性ILD，因此结论外推要慎重；（3）ILD进展主要依赖高年资专科医师进行判断，存在人为主观因素，而且在鉴别CIP与ILD影像进展方面也是难点。今后，仍需继续扩大样本量，进一步挖掘重要的临床标志物，开展大规模的多中心的前瞻性队列研究。

总之，LC-ILD患者的肺癌有着不同于单纯肺癌者的临床特征，治疗面临着风险高、预后差的挑战。治疗前需充分评估胸部HRCT、肺功能（尤其弥散功能），鉴别ILD类型，必要时MDT讨论，权衡疗效及安全性。谨慎采取个体化的肿瘤治疗以及ILD治疗方案，例如尽早开始抗纤维化治疗等。在整个治疗过程中严密监测患者的呼吸症状和影像学变化，早期发现不良反应、及时处理，从而最终改善患者预后。
